# Ameliorative Effects and Mechanism of Buyang Huanwu Decoction on Pulmonary Vascular Remodeling: Network and Experimental Analyses

**DOI:** 10.1155/2021/4576071

**Published:** 2021-08-13

**Authors:** Yucai Chen, Lidan Cui, Can Wang, Jianing Liu, Jian Guo

**Affiliations:** ^1^School of Traditional Chinese Medicine, Beijing University of Chinese Medicine, Beijing 100029, China; ^2^School of Chinese Pharmacy, Beijing University of Chinese Medicine, Beijing 100029, China

## Abstract

Pulmonary hypertension (PH) is a severe and progressive cardiovascular disease. Its pathological mechanism is complex, and the common pathological feature is pulmonary vascular remodeling. The efficacy of existing therapeutic agents is limited. Traditional Chinese medicine (TCM) has its unique advantages in the prevention and treatment of complex diseases. In this study, the approaches of network pharmacology combined with biological verification are employed to explore the role of Buyang huanwu decoction (BYHWD) in the treatment of PH. The active ingredients in BYHWD were first screened based on the ADME properties of the compounds. In turn, the mean of data mining was utilized to analyze the potential targets of BYHWD for the treatment of PH. On this basis, a series of interaction networks were constructed for searching the core targets. The genes including AKT1, MMP9, NOS3/eNOS, and EGFR were found to be possible key targets in BYHWD. The results of enrichment analysis showed that the targets of BYHWD focused on smooth muscle cell proliferation, migration, and apoptosis, which are classic biological processes involved in pulmonary vascular remodeling and are closely related to the PI3K-Akt-eNOS pathway. The methods of biological experiments were adopted to verify the above results. The present study elucidated the mechanism of BYHWD in the treatment of PH and provided new ideas for the clinical use of TCM in the treatment of PH.

## 1. Introduction

Pulmonary hypertension (PH) is a progressive vascular disease that affects the small pulmonary arteries and is characterized by heightened proliferation and apoptosis resistance of pulmonary arterial smooth muscle cells (PASMCs) [1]. Elevated pulmonary vascular resistance results in increased pulmonary arterial pressure, right ventricular failure, and ultimately death [2]. Current therapeutics for PH in clinic mainly include endothelin receptor antagonists, prostacyclin analogs, and phosphodiesterase-5 (PDE-5) inhibitors [3]. The aetiology of PH is complex, involving vasoconstriction/diastolic imbalance, gene mutation, in situ thrombosis, inflammatory response, and oxidative stress [4]. However, most drugs highlight the effect on one kind of cell, one cytokine, or one gene, without considering the complex process of PH [5].

Various kinds of complementary and/or alternative medicines have attracted growing interest in the long-term treatment of complex diseases with approved curative therapeutic effects. Relevant studies have shown that the traditional Chinese medicine (TCM) and active natural products generate attractive effects on PH treatment and rehabilitation. Maxingxiongting mixture, composed of four herbs, could attenuate hypoxia-induced PH and improve right ventricular hypertrophy by inhibiting the rho-kinase signaling pathway [6]. The Qishen Yiqi formula may play a therapeutic role in PH through the PI3K-Akt, MAPK, and HIF-1 signaling pathways [7]. The Qiliqiangxin formula could inhibit PH-induced right ventricular remodeling by reducing mitochondrial-related apoptosis pathway and reversing mitochondrial-related metabolic transition [8]. Therefore, the TCM has its unique advantages in the treatment of complex diseases such as PH because of its multicomponent, multitarget, and multipathway mechanism characteristics. It is particularly important to explore the underlying mechanisms of these TCM formulas that have been widely used and are highly effective in the clinic for their promotion and optimization. But just using traditional biological experimental methods to explore the mechanism of TCM has its limitation. Network pharmacology provides a new way to solve this problem. By constructing the compounds-targets-diseases network as well as the subsequent topological parameter analysis, the key targets and signaling pathways through which drugs exert their effects can be extracted from the complex interaction relationships, providing new ideas for investigating the multitarget drug treatment as well as mechanism [9, 10].

Buyang huanwu decoction (BYHWD) is a well-known traditional Chinese medicine formula. The BYHWD is composed of seven kinds of Chinese medicine: Huangqi (Radix Astragali seu Hedysari), Danggui (Radix Angelicae Sinensis), Chishao (Radix Paeoniae Rubra), Chuanxiong (Rhizoma Ligustici Chuanxiong), Taoren (Semen Persicae), Honghua (Flos Carthami), and Dilong (Pheretima), and all of which are recorded in the Chinese Pharmacopoeia. Clinical and preclinical studies indicate the protective functions of BYHWD on cardiocerebrovascular disease [11–13]. Despite its clinical effectiveness, the mechanism of BYHWD on pulmonary vascular diseases has not been fully investigated.

**Figure 1:**
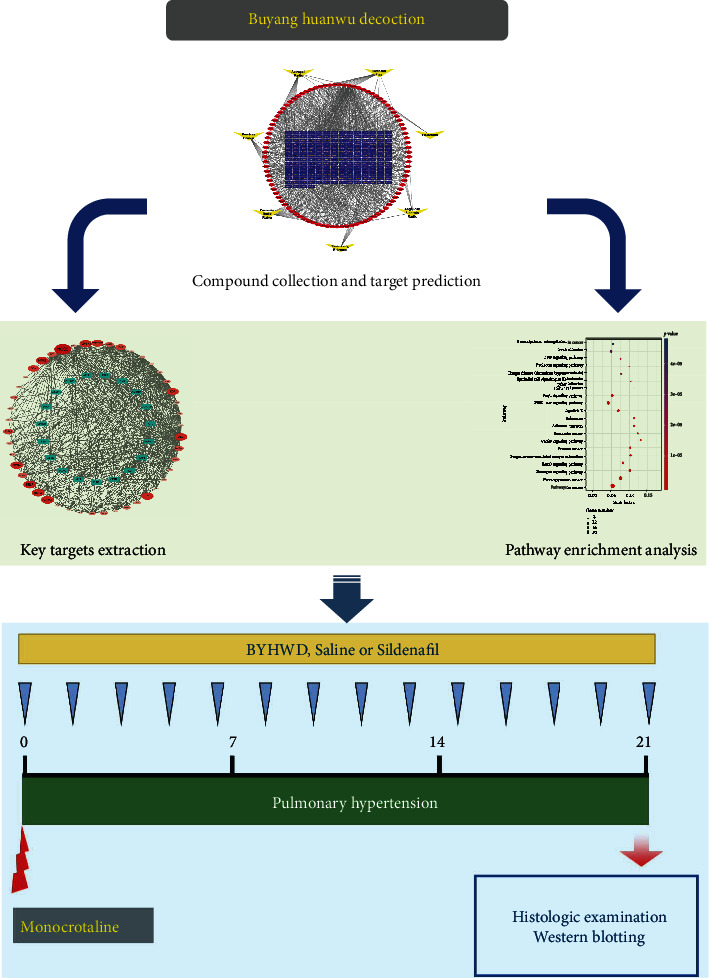
A schematic diagram of the integrative strategy combining network pharmacology analysis and biological experiments for investigation of the effect and mechanisms of BYHWD against PH.

In the present study, network pharmacology approaches were employed to investigate potential pharmacological mechanisms of BYHWD as a therapy for PH. Targets of compounds in BYHWD and PH-related genes were acquired from public databases. Then, the protein-protein interaction (PPI) was obtained to analyze the key targets, and Gene Ontology (GO) and Kyoto Encyclopedia of Genes and Genomes (KEGG) enrichment was performed to find the potential mechanism of BYHWD against PH. In addition, *in vivo* experiments were conducted to verify the key targets and pathway predicted in the network.  Figure 1 illustrates the workflow.

## 2. Materials and Methods

### 2.1. Screening the Components of BYHWD and Predicting Targets of the Components

The components of seven herbs were collected through the traditional Chinese medicine systems pharmacology platform (TCMSP, http://old.tcmsp-e.com/tcmsp.php) and TCM Taiwan database (http://tcm.cmu.edu.tw/zh-tw/) [14, 15]. Oral bioavailability (OB), Caco-2 permeability, and drug like availability (DL) are important pharmacokinetic parameters to evaluate the drugability of compounds in the ADME process. The parameters of active compounds for selection were set as follows: OB > 30%, DL > 0.18, and Caco‐2 > −0.4 [16]. Some compounds were extremely high in content or were reported to have strong pharmacological activities; then, they were included in further evaluation, even if they did not completely meet the above screening principles. Totally, we collected 93 compounds in BYHWD. The detailed information about chemical constituents of each herb in BYHWD is showed in Table S1. The obtained Mol2 type files of ingredients were retrieved using the TCMSP database. Next, potential targets were predicted by importing each Mol2 formatted file into the Pharmmapper online tool (http://www.lilab-ecust.cn/pharmmapper/) [17]. Based on the normalized fit score, the top 10 highly matching targets of each were selected. In addition, the SMILES structural information of compounds was obtained through the PubChem database, and targets with a match score greater than 0.5 for each compound were retrieved in the SwissTargetPrediction database based on the principle of structural similarity (http://www.swisstargetprediction.ch/) [18].

### 2.2. Collecting the PH-Related Targets

With “pulmonary hypertension” as the keyword, the genes associated with PH were collected from the DisGeNET database (http://www.disgenet.org) and the Comparative Toxicogenomics Database (http://ctdbase.org/) [19]. Duplicate genes in the search results were discarded. The UniProt database (http://www.uniprot.org/) was used to acquire the standard gene symbol with the organism selected as “Homo sapiens.” Finally, a total of 901 genes associated with PH was obtained. The intersection of targets of active components and PH-related targets was considered to be potential therapeutic targets of BYHWD against PH. The details are described in Table S2.

### 2.3. Construction of the PPI Network

The STRING database (https://www.string-db.org/) includes both direct physical interactions and indirect functional correlations between proteins. It contains not only experimental data, results from PubMed abstracts, and other database data but also the results predicted by bioinformatics methods [20]. Using this platform, the interaction relationship network among potential targets of BYHWD was constructed. Network visualization was performed using Cytoscape 3.8.2. Topological parameters of these nodes such as the degree, betweenness centrality, closeness centrality, and average shortest path were analyzed by Network Analyzer in Cytoscape software. Among them, the degree value, one of the most predominant topological parameters in the network, was used to characterize the degree of nodes. The larger the value of the degree parameter of a node, the more central the node was considered in the topological network.

### 2.4. Gene-Phenotype Correlation Analysis

The VarElect online platform (https://varelect.genecards.org/), which is an integral part of the GeneCards database, can analyze the correlation of a large number of input genes with a certain clinical phenotype [21]. The input genes were divided into phenotype-directly related genes and phenotype-indirectly related genes, and the score of correlation was obtained. According to VarElect's calculation method, the correlation score in the result is derived from the frequency of a specific phenotype in individual GeneCards. The higher the score, the higher the correlation between the gene and a specific phenotype [21]. In this study, this tool was used to analyze the correlation between the potential targets of BYHWD and the phenotype of “pulmonary hypertension.”

### 2.5. GO and KEGG Enrichment

The Database for Annotation, Visualization, and Integrated Discovery (DAVID, https://david.ncifcrf.gov/home.jsp, ver. 6.8) was applied for GO and KEGG enrichment analysis [22]. The potential therapeutic targets of BYHWD against PH were input into the DAVID platform, and the species was set as “Homo sapiens.” The biological processes (BP), molecular functions (MF), and KEGG pathway enrichment analysis were carried out, respectively. Only GO terms and KEGG pathways with *P* values < 0.01 were included. Not only that, the potential therapeutic targets of BYHWD against PH were entered into the KEGG Mapper (https://www.genome.jp/kegg/mapper.html) for further analysis of genes included in key pathways. Based on these results, the top 20 terms with the smallest *P* value and their parameters are uploaded to Omicshare (https://www.omicshare.com/) online tools for further visual analysis.

### 2.6. PH Animal Model and Drug Treatment

The adult male Sprague-Dawley rats (180–220 g) were supplied by the Vital River Laboratory Animal Center (Beijing, China). The animals were housed under a 12 h light-dark cycle at the condition with a temperature at 21–25°C and the humidity of 55%–65%. The rats were allowed access to water and commercial food. All the animal procedures were performed in accordance with the *Guide for the Care and Use of Laboratory Animals* (National Institutes of Health (NIH), USA). The monocrotaline (MCT) was purchased from Sigma-Aldrich, St. Louis, MO. The sildenafil was purchased from Yuanye Bio-Technology Co., Ltd (Shanghai, China). The BYHWD concentrated granules were obtained from Beijing Tcmages Pharmaceutical Co., Ltd (Beijing, China). Rats in the MCT-induced PH groups received a single subcutaneous injection of MCT (50 mg/kg) on day 1. The animals were further treated with either saline (MCT group), BYHWD solution (20 g/kg daily for 21 days, the BYHWD groups), or sildenafil solution (20 mg/kg daily for 21 days, the sildenafil groups, as the positive drug group). The control group was treated with saline but did not receive MCT injection.

### 2.7. Histologic Examination

By the experimental endpoint, the rats were euthanized. The left lung was removed and formalin-fixed and paraffin-embedded. Serial sections (5 *μ*m) were routinely stained using Masson's trichrome staining. Digital images were obtained at 200x magnification by microscopy (Olympus, Tokyo, Japan). The medial wall thickness was measured as described previously [23].

### 2.8. Western Blot

To separate the protein of lungs, the left lung samples were homogenized with whole lysis buffer (containing 0.02 mol/L Tris-HCl, 1% Triton X-100, 0.15 mol/L NaCl, 1 mmol/L ethylenediamine tetraacetic acid, 1 mmol/L EGTA, 2.5 mmol/L sodium pyrophosphate, 1 mmol/L *β*-glycerophosphate, and 1 mmol/L sodium orthovanadate). Western blot assays of PI3K, Akt, and eNOS were carried out by standard protocols based on the previous study [24]. In brief, the supernatants were fractionated by 8% or 10% SDS-PAGE and then electrotransferred onto a PVDF membrane. The membranes were blocked with 5% BSA in Tris-buffered saline plus 0.1% Tween-20 (TBST) for 1 h at 37°C. Then, they were subjected to an immunoblotting assay using primary antibodies to PI3K (Abcam, ab40755, 1 : 1000), phospho-Akt (Abcam, ab81283, 1 : 1000), total-Akt (Abcam, ab179463, 1 : 2000), and eNOS (CST, 32027, 1 : 1000) as well as *β*-actin (Proteintech, 66009, 1 : 2000) as an internal reference. After incubation with a properly titrated second antibody, the immunoblot on the membrane was visible after development with an enhanced chemiluminescence (ECL) system.

### 2.9. Statistical Analysis

Data are expressed as the mean ± SEM of at least three independent experiments and analyzed by GraphPad Prism 7 (GraphPad Software, San Diego, CA, USA). Statistical analysis was performed using one-way analysis of variance (ANOVA) followed by Dunnett's multiple comparison test. A *P* value less than 0.05 was regarded as significant.

## 3. Results

### 3.1. Screening of Active Ingredients and Targets of BYHWD

**Figure 2:**
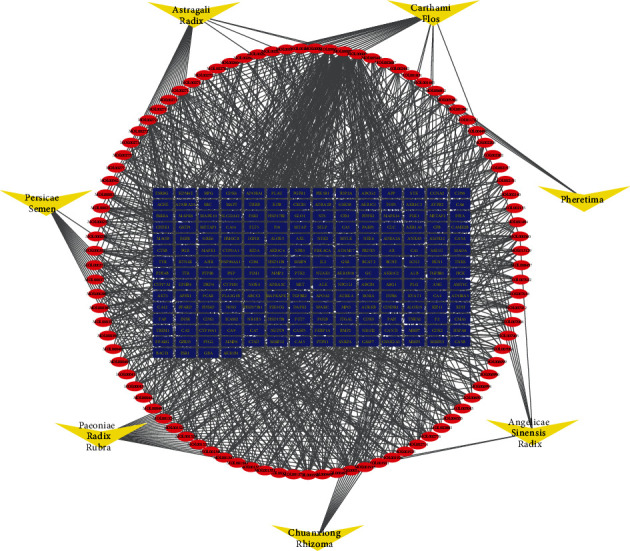
Global herbs-compounds-targets network in the BYHWD. Yellow nodes represent herb components in the formula, 93 red nodes represent compound components in each herb, 200 blue nodes remark the targets of BYHWD from prediction and literature mining, and edges represent interactions between them.

According to the properties of compounds such as OB and DL, 93 components were screened out from seven herbs. Part of the compounds, such as ferulic acid (MOL000360), chrysanthemaxanthin (MOL004492), and stigmasterol (MOL000449), are present simultaneously in multiple herbs. Based on structural similarity prediction and reverse molecular docking, a total of 200 predicted targets of the compounds were collected (Figure 2). The above results partly illustrate that there is a common effect for the individual constituent components in the formula.

### 3.2. Interaction Analysis of Anti-PH-Related Ingredients and Targets in BYHWD

**Figure 3:**
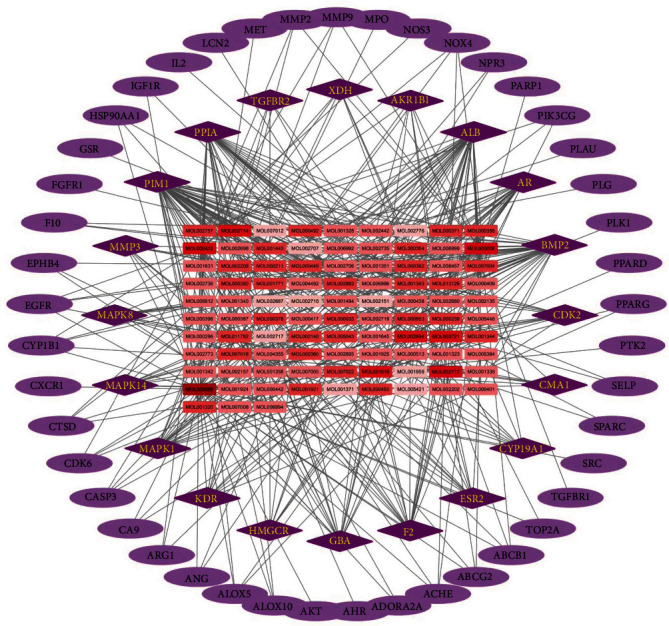
Specific compounds-targets network of ingredients against PH in the BYHWD. The purple nodes represent the potential therapeutic targets of BYHWD against pH, among which the 20 nodes in dark purple were the core nodes in the network. The red square nodes remark the compounds that are linked to the therapeutic targets, and the darker the red color, the higher its degree value in the network.

Through further analysis, 65 targets associated with PH among all targets of BYHWD were obtained. Ingredients in BYHWD associated with these 65 targets were acquired. The compounds-targets network was constructed by Cytoscape software. As shown in Figure 3, the C-T network consists of 158 nodes (93 compounds and 65 PH-related targets) and 904 C-T interactions. In order to find the main active compounds, the topological parameters of the network were further analyzed. The results indicated that the compounds with the highest degree parameter in the network included quercetin, kaempferol, luteolin, 6-hydroxykaempferol, quercetagetin, and baicalein. These compounds may play a more important role in the anti-PH effects of BYHWD.

### 3.3. Constructing PPI Networks and Analyzing Network Topological Features

**Figure 4:**
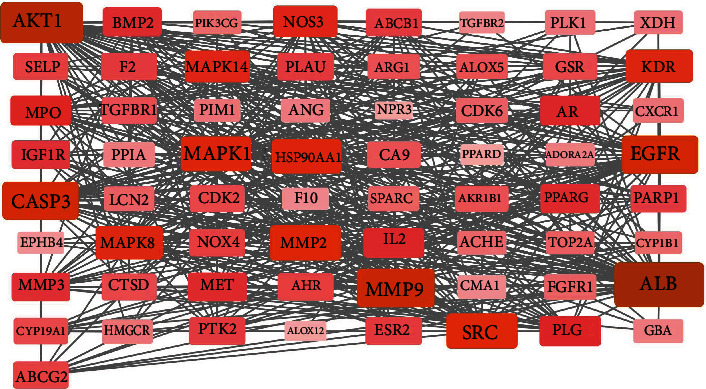
Network biology analysis for PPI constructed with potential targets of BYHWD against PH. In the network, nodes represent targets. Edges (gray) represent PPI interactions. The color intensity of a node is proportional to the value of a degree in the network.

The degree is one of the important topological parameters, which equals the number of links connected to and also the number of neighbors of the node. The value of the degree reflects the importance of the node in the network. Using the STRING online tool, the PPI network of BYHWD's potential targets for the treatment of PH was constructed. As shown in Figure 4, the targets with higher degree values in the network included AKT1, ALB, and MMP9. These key targets were more interconnected with the other targets and were also involved in multiple signaling pathways. So, these core targets may play a more critical role in the anti-PH effects of BYHWD.

### 3.4. Gene-Phenotype Correlation Analysis of the Targets of BYHWD

**Figure 5:**
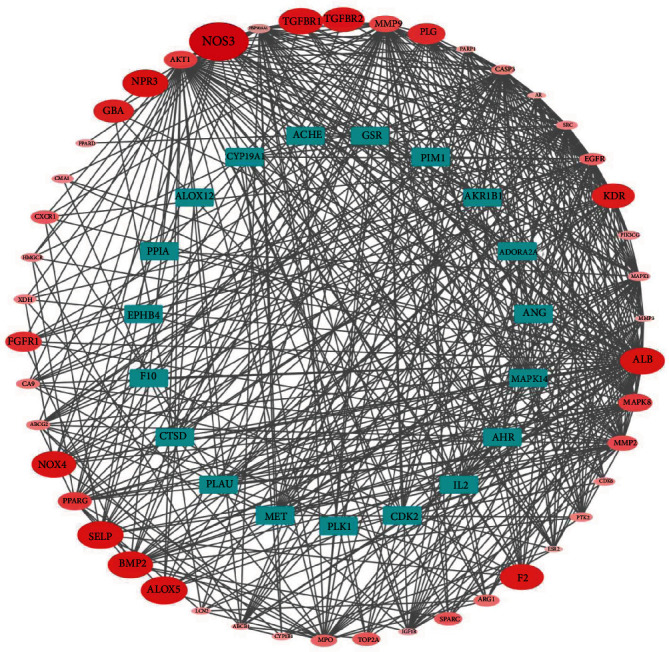
Correlation between target genes and the PH phenotypes. Green nodes located on the inner circle represent genes that are indirectly associated with PH. The red nodes located on the outer ring represent the directly related genes. The degree of darkness of node color is proportional to its phenotypic relevance.

PH is a kind of disease with complex pathogenesis. A large number of genes are implicated in the development and progression of PH. These genes vary in the level of significance of the role they are involved in. With the VarElect online tool, potential targets to the PH phenotype correlations were analyzed. Among the 65 analyzed genes, 46 were directly associated with the disease phenotype and 19 were indirectly associated (Figure 5). Among the genes directly associated with PH, the 10 most strongly associated genes were NOS3, ALOX5, SELP, NPR3, BMP2, ALB, NOX4, TGFBR1, F2, and KDR (Table 1). Many of these genes also coincide with the core genes in the PPI network.

### 3.5. Gene Ontology Analysis

**Figure 6:**
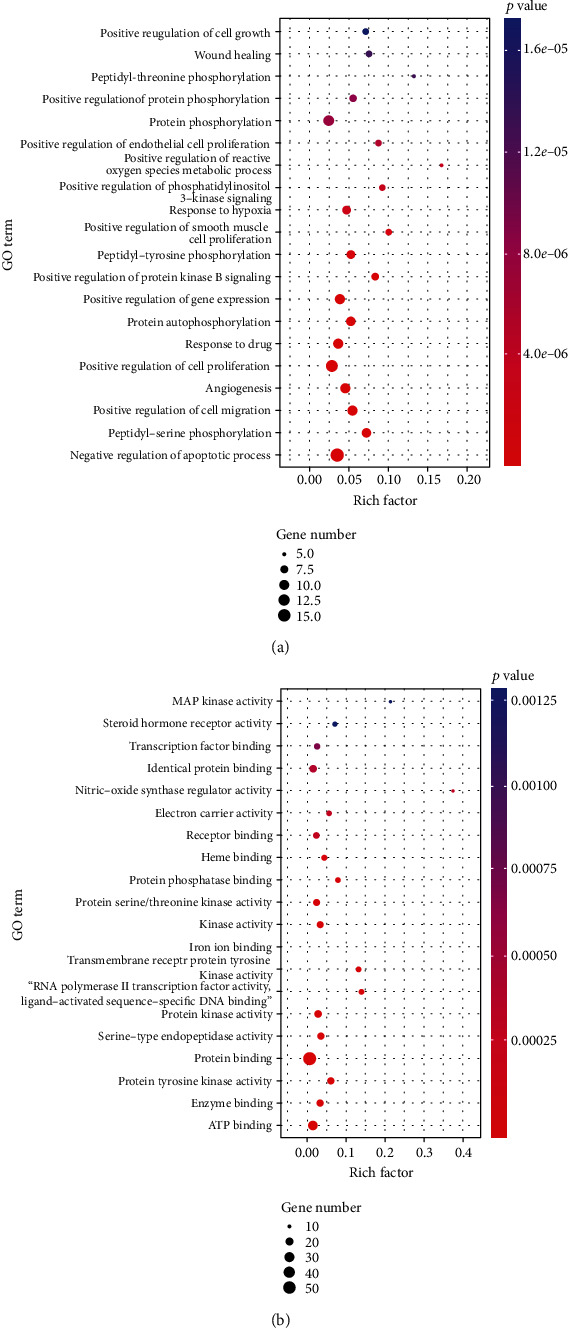
GO enrichment analysis map, containing (a) biological process and (b) molecular function. The size of the dots indicated the number of enriched targets, and the color of the dots represented the degree of significance based on the *P* value.

The enrichment analysis of potential targets of BYHWD in the treatment of PH was performed using the DAVID platform to explore the BP and MF in which these targets were involved. As shown in Figure 6 and Tables S3 and S4, potential therapeutic targets are involved in many biological processes, including “negative regulation of apoptotic process,” “positive regulation of cell migration,” “angiogenesis,” “positive regulation of cell promotion,” “positive regulation of protein kinase b signaling,” “positive regulation of smooth muscle cell promotion,” “response to hypoxia,” and “positive regulation of phosphatidylinositol 3-kinase signaling.” The molecular function terms involved in these targets included “ATP binding,” “enzyme binding,” “protein tyrosine kinase activity,” and “protein binding.” Obviously, many of the biological processes enriched were closely related to the mechanism of vascular remodeling in PH.

### 3.6. Pathway Enrichment Analysis

**Figure 7:**
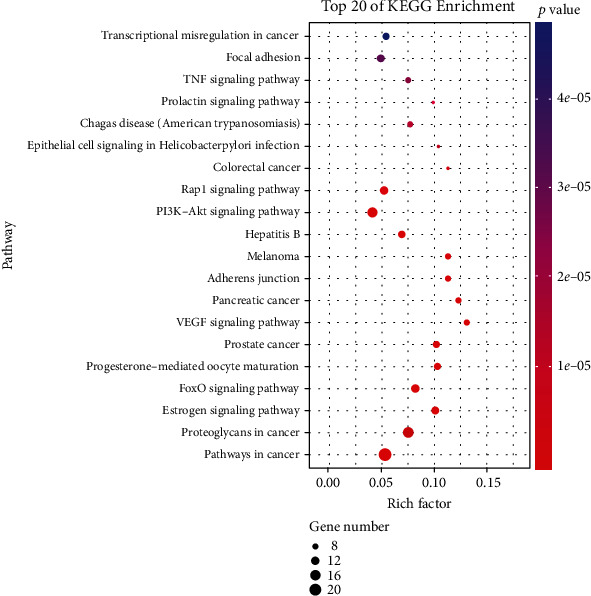
The KEGG analysis for the potential targets of BYHWD against PH. The color represents the different *P* values (<0.05), while the size of the circle represents the count.

**Figure 8:**
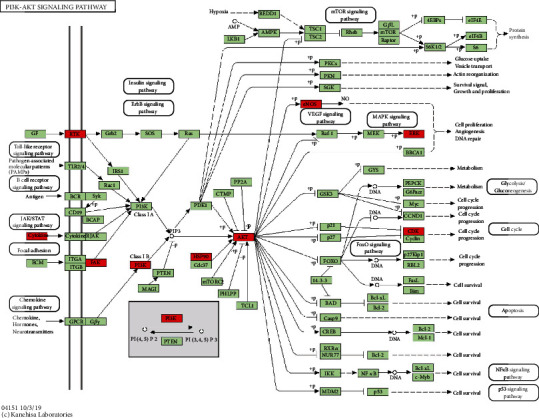
Distribution of the target proteins of BYHWD on the PI3K-Akt pathway. The red nodes are potential target proteins of BYHWD against PH, while the green nodes are relevant targets in the pathway.

To further clarify the relationship between targets and the pathways, as described previously, the KEGG databases and the KEGG Mapper online tool were used for pathway enrichment analysis. The top 20 pathways involving 65 targets were screened. The results showed that the targets were mainly involved in the FoxO signaling pathway, VEGF signaling pathway, PI3K-Akt signaling pathway, TNF signaling pathway, and so on (Figure 7 and Figure 8).

### 3.7. Experimental Validation

**Figure 9:**
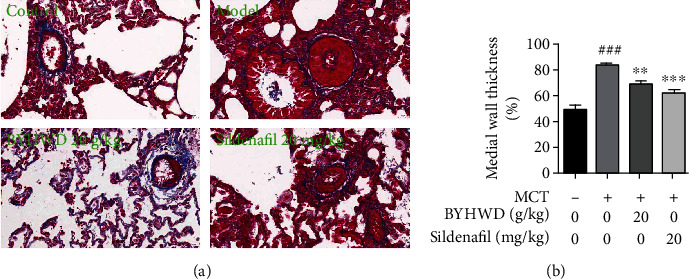
BYHWD prevents the development of small pulmonary artery hypertrophy in MCT-treated rats. (a) Representative images of the lung sections for intrapulmonary arteries stained with Masson's trichrome (original magnification ×200). (b) Medial wall thickness (%) of intrapulmonary arteries was calculated and shown in the bar graph. The results were expressed as the mean ± SEM of three independent experiments (*n* = 3). ^###^*P* < 0.001 vs. the control group; ^∗∗^*P* < 0.01 and ^∗∗∗^*P* < 0.001 vs. the model group.

**Figure 10:**
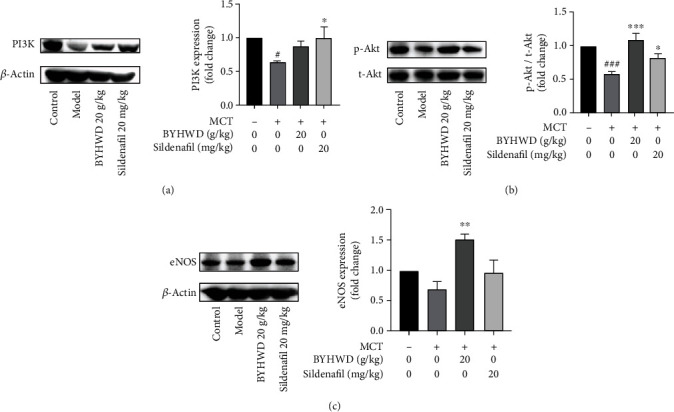
Effects of BYHWD on the PI3K/Akt/eNOS pathway. Expression of PI3K and eNOS and phosphorylation of Akt were measured by western blot analysis. Data are expressed as *X*-fold induction compared to normal control. The results were expressed as the mean ± SEM (*n* = 3-4). ^#^*P* < 0.05 and ^###^*P* < 0.001 vs. the control group; ^∗^*P* < 0.05, ^∗∗^*P* < 0.01, and ^∗∗∗^*P* < 0.001 vs. the model group.

To further investigate the effect of BYHWD on pulmonary vascular remodeling, a MCT-induced PH model was constructed. The Masson trichrome staining was used to observe the thickness of small pulmonary vessels. Pulmonary vessels with a diameter less than 150 *μ*m were included to calculate vessel wall thickening. The results showed that the thickness of pulmonary small vessels was significantly increased after administration of MCT and was significantly decreased by both BYHWD treatment and positive drug sildenafil treatment (Figure 9). According to the above mechanistic analysis, the eNOS and PI3K-Akt pathway may be the key targets of BYHWD in the treatment of PH. The approach of the biological experiment was taken to validate the obtained key targets and pathways. The results of western blot showed that the activation of the PI3K-Akt pathway was significantly reduced and the expression level of eNOS was decreased in MCT-treated rats. After administration with BYHWD, the levels of the PI3K-Akt-eNOS pathway were significantly upregulated (Figure 10).

## 4. Discussion

The prevalence of PH and the incapability of existing vasodilation therapy in confronting the complex feature disorder make the present treatment strategies controversial. TCM formulas produce synergistic effects by potentially active ingredients acting in a multitarget and multipathway biological network, thereby interfering with the occurrence and development of diseases and finally achieving therapeutic effects. BYHWD has been widely used in the treatment of many cardiovascular and cerebrovascular diseases. A recent study reported that BYHWD could promote neurological recovery and after intracerebral hemorrhage by activating VEGFR2 through the PI3K/Akt signaling pathway [11]. The active components of BYHWD have multitarget neuroprotective effects on ischemic stroke, and its mechanism may be closely related to the improvement of blood supply [25]. A study based on a tag-based digital gene expression profiling (DGE) system showed that the mechanism of ventricular remodeling induced by myocardial infarction by BYHWD treatment may be closely related to transforming growth factor beta receptor-1, junctophilin-2, and monocyte [26]. In this study, network pharmacology-based approaches were conducted to explore the underlying mechanism of BYHWD in PH.

Firstly, we predicted the potential targets of BYHWD for the treatment of PH combining potential active ingredients, putative targets of compounds in BYHWD, and targets of PH. Through constructing the herbs-compounds-targets network as well as analyzing topological parameters, the active compounds in BYHWD for the treatment of PH were extracted, including quercetin, kaempferol, luteolin, 6-hydroxykaempferol, quercetagetin, and baicalein. Many studies have reported the anti-PH effects of these compounds *in vitro* and *in vivo*. Studies demonstrate that quercetin can inhibit pulmonary arterial endothelial cell transdifferentiation into smooth muscle-like cells by regulating Akt and ERK1/2 pathways and enhance the autophagy of PASMCs induced by hypoxia through the FoxO1-SENS3-mTOR pathway [27, 28]. Quercetin can also interfere with the IRE1*α*-XBP1 pathway and TrkA/kt pathway, leading to reduced PASMC migration and cell cycle arrest, which in turn ameliorates hypoxia-induced PH [29, 30]. Luteolin can inhibit PDGF-induced proliferation and migration of PASMCs in a dose-dependent manner via regulating the Hippo-YAP/PI3K/Akt signaling pathway [31]. The role of baicalein in the treatment of PH is the focus of numerous investigators. Baicalein can delay the progression of pulmonary vascular remodeling by inhibiting the NF-*κ*B pathway and the catenin/ET-1/ETR pathway [32, 33]. Baicalein also could attenuate MCT-induced PH by inhibiting endothelial-to-mesenchymal transition [34]. The above results suggest that numerous active ingredients in BYHWD exert effects against pulmonary vascular remodeling by regulating multiple signal transduction pathways.

Furthermore, the PPI network and gene-disease phenotype correlation of potential therapeutic targets were analyzed to find the core targets of BYHWD. AKT1, MMP9, NOS3/eNOS, and EGFR may play critical roles in the anti-PH effects of BYHWD. Impaired availability of NO is a key underlying feature in most forms of PH. NO is produced catalytically from L-arginine by the PI3K-Akt-eNOS pathway and regulates pulmonary vascular function and structure. The decrease of eNOS expression and dysfunction will lead to the inhibition of the NO signal in the lung and the disruption of pulmonary vasoconstriction/relaxation balance [35]. Dacomitinib, an EGFR inhibitor, can significantly inhibit vascular thickening, collagen deposition, and right ventricular remodeling in PH rats [36]. Many studies have shown that matrix metalloproteinases (MMP) play a key role in the disease state, especially in extracellular matrix remodeling and vascular homeostasis [37]. Increased expression of extracellular matrix proteins is closely associated with MMP activation in pulmonary arteries [38]. There are also close interactions between these targets. Vascular remodeling progression involves cell migration and invasion as well as protein degradation of the ECM in which it participates. Upon activation of the PI3K/Akt pathway, phosphorylated Akt can further activate MMP9, thereby playing a role in cell migration and invasion [39]. Dacomitinib could inhibit cell cycle progression, proliferation, and migration and enhance cell autophagy in PASMCs induced by hypoxia via the PI3K-Akt-mTOR signaling pathway [36].

The approaches of enrichment analysis were further adopted to analyze the biological processes and signaling pathways involved in potential targets of BYHWD. Multiple canonical biological processes closely associated with PH, such as “negative regulation of apoptotic process,” “positive regulation of cell migration,” “angiogenesis,” “positive regulation of smooth muscle cell proliferation,” and “positive regulation of phosphatidylinositol 3-kinase signaling,” were discovered in the study. The KEGG enrichment results showed that the signaling pathways that play therapeutic roles by BYHWD may include “VEGF signaling pathway,” “PI3K-Akt signaling pathway,” “TNF signaling pathway,” and “FoxO signaling pathway.” VEGF is one of the strongest activators of angiogenesis, which stimulates the migration and proliferation of vascular endothelial cells, thereby promoting angiogenesis. There is a close relationship between the overexpression of VEGF and the pathological process of PH. Under hypoxic conditions, VEGF is released under the regulation of hypoxia-inducible factor (HIF-1), which in turn causes pulmonary vascular inflammation [40]. Akt mediates cell proliferation induced by various growth factors, which have been widely demonstrated. As an upstream regulatory pathway, Akt could be activated by several stimuli, including VEGF and PDGF. VEGF-induced endothelial migration is mediated through the Akt pathway. In terms of vascular smooth muscle cell apoptosis, FoxOs could induce the expression of the proapoptotic Bcl-2 family of proteins or upregulated the expression of death receptor ligands such as Fas ligand and TNF*α*. Akt can promote the phosphorylation of FoxO, which in turn inhibits the transcriptional function of FoxO, downregulates Bcl-2 levels, and regulates cell apoptosis [41]. It can be seen that BYHWD could exert therapeutic effects on PH by acting on multiple targets and multiple signaling pathways. While in the analysis of topological parameters of the PPI network, it was found that Akt was in the central position in the target network. The results of gene-phenotype correlation analysis suggest that NOS3/eNOS may be one of the most critical genes in which BYHWD functions. As can be seen in the GO enrichment analysis results, the potential targets of BYHWD in the treatment of PH were significantly enriched in “positive regulation of protein kinase b signaling” and “positive regulation of phosphatidylinositol 3-kinase signaling.” And in the KEGG enrichment analysis results, the term “PI3K-Akt signaling pathway” also appeared. As mentioned above, there is also a tight connection between the PI3K-Akt-eNOS pathway and other core potential targets, such as VEGF and MMP2. Based on the above experimental results of core target analysis and enrichment analysis, combined with previous studies, we speculate that the PI3K-Akt-eNOS pathway may be one of the crucial signaling pathways through which BYHWD exerts therapeutic effects on PH.

On the basis of network pharmacology analysis, the MCT-induced PH model was then constructed to preliminarily explore the efficacy and verify the mechanism of BYHWD in treating PH. Masson's trichrome staining results showed that the thickening of the pulmonary small vessel was noticeable after rats were stimulated with MCT for 21 days. BYHWD treatment can significantly inhibit the vascular remodeling process, which illustrates that BYHWD could ameliorate the PH development from the key pathological mechanism. This is also consistent with the conclusion from network pharmacology analysis that BYHWD may exert effects against PH by regulating the proliferation, apoptosis, and migration of vascular smooth muscles. Network pharmacology results revealed that the PI3K-Akt-eNOS signaling pathway may play a central role in the therapeutic effects of BYHWD. Then, western blotting was designed to validate the changes of the PI3K-Akt-eNOS pathway in lung tissues of PH rats and the effects of BYHWD treatment. Results indicated that BYHWD increased the expression and the phosphorylation of Akt. Collectively, BYHWD repressed pulmonary arterial remodeling partially by modulating the expression of the PI3K-Akt-eNOS pathway.

## 5. Conclusion

In conclusion, the present study demonstrated that BYHWD can exert its effects on inhibiting pulmonary vascular remodeling through multitargets and multipathways by the approach of network pharmacology as well as biological experimental verification. The most prominent mechanism may be related to the activation of the PI3K-Akt-eNOS pathway.

## Figures and Tables

**Table 1:** Top 10 targets directly associated with PH phenotype.

Gene symbol	Description	Score
NOS3	Nitric oxide synthase 3	21.08
ALOX5	Arachidonate 5-lipoxygenase	14.37
SELP	Selectin P	14.25
NPR3	Natriuretic peptide receptor 3	13.96
BMP2	Bone morphogenetic protein 2	13.68
ALB	Albumin	13.56
NOX4	NADPH oxidase 4	13.42
TGFBR1	Transforming growth factor beta receptor 1	13.32
F2	Coagulation factor II, thrombin	12.99
KDR	Kinase insert domain receptor	12.33

## Data Availability

The data used to support the findings of this study are available from the corresponding authors upon request.

## Supplementary Materials

Supplementary Materials Table S1: 93 compounds in BYHWD. Table S2: the potential therapeutic targets of BYHWD against PH. Table S3: the result of top 20 GO (biological process) enrichment analysis. Table S4: the result of top 20 GO (molecular function) enrichment analysis.
