# Supplementary material for: Ameliorative Effects and Mechanism of Buyang Huanwu Decoction on Pulmonary Vascular Remodeling: Network and Experimental Analyses
Source: Oxid Med Cell Longev. 2021 Aug 13;2021:4576071. doi: 10.1155/2021/4576071 (PMC8378953; doi:10.1155/2021/4576071)
Supplement: Supplementary Materials — Table S1: 93 compounds in BYHWD. Table S2: the potential therapeutic targets of BYHWD against PH. Table S3: the result of top 20 GO (biological process) enrichment analysis. Table S4: the result of top 20 GO (molecular function) enrichment analysis. [file 4576071.f1.docx]

Table S1 93 compounds in BYHWD

| **Mol ID** | **Molecule Name** | **Herb** | **MW** | **OB (%)** | **Caco-2** | **DL** |
| --- | --- | --- | --- | --- | --- | --- |
| MOL000360 | ferulic acid | Angelicae Sinensis Radix | 194.2 | 39.56 | 0.47 | 0.06 |
| MOL011782 | Ligustilide | Angelicae Sinensis Radix | 190.26 | 23.5 | 1.28 | 0.07 |
| MOL001956 | Cnidilin | Angelicae Sinensis Radix | 300.33 | 32.69 | 1.03 | 0.28 |
| MOL006812 | Phyllanthin | Angelicae Sinensis Radix | 418.58 | 33.31 | 1.06 | 0.42 |
| MOL005384 | Suchilactone | Angelicae Sinensis Radix | 368.41 | 57.52 | 0.82 | 0.56 |
| MOL004492 | Chrysanthemaxanthin | Angelicae Sinensis Radix | 584.96 | 38.72 | 0.51 | 0.58 |
| MOL000358 | beta-sitosterol | Angelicae Sinensis Radix | 414.79 | 36.91 | 1.32 | 0.75 |
| MOL000449 | Stigmasterol | Angelicae Sinensis Radix | 412.77 | 43.83 | 1.44 | 0.76 |
| MOL000360 | ferulic acid | Astragali Radix | 194.2 | 39.56 | 0.47 | 0.06 |
| MOL000409 | AstragalosideIV | Astragali Radix | 785.09 | 17.74 | -2.22 | 0.15 |
| MOL000401 | Astragaloside I | Astragali Radix | 707.01 | 12.34 | -1.15 | 0.2 |
| MOL000392 | formononetin | Astragali Radix | 268.28 | 69.67 | 0.78 | 0.21 |
| MOL000422 | kaempferol | Astragali Radix | 286.25 | 41.88 | 0.26 | 0.24 |
| MOL000417 | Calycosin | Astragali Radix | 284.28 | 47.75 | 0.52 | 0.24 |
| MOL000438 | (3R)-3-(2-hydroxy-3,4-dimethoxyphenyl)chroman-7-ol | Astragali Radix | 302.35 | 67.67 | 0.96 | 0.26 |
| MOL000098 | quercetin | Astragali Radix | 302.25 | 46.43 | 0.05 | 0.28 |
| MOL000239 | Jaranol | Astragali Radix | 314.31 | 50.83 | 0.61 | 0.29 |
| MOL000398 | isoflavanone | Astragali Radix | 316.33 | 109.99 | 0.53 | 0.3 |
| MOL000378 | 7-O-methylisomucronulatol | Astragali Radix | 316.38 | 74.69 | 1.08 | 0.3 |
| MOL000354 | isorhamnetin | Astragali Radix | 316.28 | 49.6 | 0.31 | 0.31 |
| MOL000380 | (6aR,11aR)-9,10-dimethoxy-6a,11a-dihydro-6H-benzofurano[3,2-c]chromen-3-ol | Astragali Radix | 300.33 | 64.26 | 0.93 | 0.42 |
| MOL000442 | 1,7-Dihydroxy-3,9-dimethoxy pterocarpene | Astragali Radix | 314.31 | 39.05 | 0.89 | 0.48 |
| MOL000371 | 3,9-di-O-methylnissolin | Astragali Radix | 314.36 | 53.74 | 1.18 | 0.48 |
| MOL004492 | Chrysanthemaxanthin | Astragali Radix | 584.96 | 38.72 | 0.51 | 0.58 |
| MOL000387 | Bifendate | Astragali Radix | 418.38 | 31.1 | 0.15 | 0.67 |
| MOL000296 | hederagenin | Astragali Radix | 414.79 | 36.91 | 1.32 | 0.75 |
| MOL000211 | Mairin | Astragali Radix | 456.78 | 55.38 | 0.73 | 0.78 |
| MOL000033 | (3S,8S,9S,10R,13R,14S,17R)-10,13-dimethyl-17-[(2R,5S)-5-propan-2-yloctan-2-yl]-2,3,4,7,8,9,11,12,14,15,16,17-dodecahydro-1H-cyclopenta[a]phenanthren-3-ol | Astragali Radix | 428.82 | 36.23 | 1.45 | 0.78 |
| MOL000360 | ferulic acid | Carthami Flos | 194.2 | 39.56 | 0.47 | 0.06 |
| MOL002736 | safflower-yellow-B | Carthami Flos | 1091.07 | 3.01 | -4.2 | 0.17 |
| MOL002757 | 7,8-dimethyl-1H-pyrimido[5,6-g]quinoxaline-2,4-dione | Carthami Flos | 242.26 | 45.75 | 0.06 | 0.19 |
| MOL002717 | qt_carthamone | Carthami Flos | 286.25 | 51.03 | -0.31 | 0.2 |
| MOL001645 | Linoleyl acetate | Carthami Flos | 308.56 | 42.1 | 1.36 | 0.2 |
| MOL002714 | baicalein | Carthami Flos | 270.25 | 33.52 | 0.63 | 0.21 |
| MOL000422 | kaempferol | Carthami Flos | 286.25 | 41.88 | 0.26 | 0.24 |
| MOL002719 | 6-Hydroxynaringenin | Carthami Flos | 288.27 | 33.23 | 0.27 | 0.24 |
| MOL000006 | luteolin | Carthami Flos | 286.25 | 36.16 | 0.19 | 0.25 |
| MOL002712 | 6-Hydroxykaempferol | Carthami Flos | 302.25 | 62.13 | 0.16 | 0.27 |
| MOL000098 | quercetin | Carthami Flos | 302.25 | 46.43 | 0.05 | 0.28 |
| MOL002721 | quercetagetin | Carthami Flos | 318.25 | 45.01 | -0.06 | 0.31 |
| MOL002698 | lupeol-palmitate | Carthami Flos | 665.26 | 33.98 | 1.52 | 0.32 |
| MOL002710 | Pyrethrin II | Carthami Flos | 372.5 | 48.36 | 0.53 | 0.35 |
| MOL002694 | 4-[(E)-4-(3,5-dimethoxy-4-oxo-1-cyclohexa-2,5-dienylidene)but-2-enylidene]-2,6-dimethoxycyclohexa-2,5-dien-1-one | Carthami Flos | 356.4 | 48.47 | 0.81 | 0.36 |
| MOL002706 | Phytoene | Carthami Flos | 545.04 | 39.56 | 2.22 | 0.5 |
| MOL002707 | phytofluene | Carthami Flos | 543.02 | 43.18 | 2.29 | 0.5 |
| MOL002680 | Flavoxanthin | Carthami Flos | 584.96 | 60.41 | 0.97 | 0.56 |
| MOL002773 | beta-carotene | Carthami Flos | 536.96 | 37.18 | 2.25 | 0.58 |
| MOL002695 | lignan | Carthami Flos | 458.55 | 43.32 | 0.42 | 0.65 |
| MOL000953 | CLR | Carthami Flos | 386.73 | 37.87 | 1.43 | 0.68 |
| MOL002735 | safflow-yellow-A | Carthami Flos | 610.57 | 27.16 | -2.9 | 0.7 |
| MOL005421 | alpha-Onocerin | Carthami Flos | 442.8 | 39.31 | 0.86 | 0.73 |
| MOL002776 | Baicalin | Carthami Flos | 446.39 | 40.12 | -0.85 | 0.75 |
| MOL000358 | beta-sitosterol | Carthami Flos | 414.79 | 36.91 | 1.32 | 0.75 |
| MOL001771 | poriferast-5-en-3beta-ol | Carthami Flos | 414.79 | 36.91 | 1.45 | 0.75 |
| MOL000449 | Stigmasterol | Carthami Flos | 412.77 | 43.83 | 1.44 | 0.76 |
| MOL002202 | tetramethylpyrazine | Chuanxiong Rhizoma | 136.22 | 20.01 | 1.19 | 0.03 |
| MOL000360 | ferulic acid | Chuanxiong Rhizoma | 194.2 | 39.56 | 0.47 | 0.06 |
| MOL011782 | Ligustilide | Chuanxiong Rhizoma | 190.26 | 23.5 | 1.28 | 0.07 |
| MOL002208 | Senkyunolide A | Chuanxiong Rhizoma | 192.28 | 26.56 | 1.3 | 0.07 |
| MOL001494 | Mandenol | Chuanxiong Rhizoma | 308.56 | 42 | 1.46 | 0.19 |
| MOL002151 | senkyunone | Chuanxiong Rhizoma | 326.52 | 47.66 | 1.15 | 0.24 |
| MOL002140 | Perlolyrine | Chuanxiong Rhizoma | 264.3 | 65.95 | 0.88 | 0.27 |
| MOL002135 | Myricanone | Chuanxiong Rhizoma | 356.45 | 40.6 | 0.67 | 0.51 |
| MOL004492 | Chrysanthemaxanthin | Chuanxiong Rhizoma | 584.96 | 38.72 | 0.51 | 0.58 |
| MOL002157 | wallichilide | Chuanxiong Rhizoma | 412.57 | 42.31 | 0.82 | 0.71 |
| MOL000513 | 3,4,5-trihydroxybenzoic acid | Paeoniae Radix Rubra | 170.13 | 31.69 | -0.09 | 0.04 |
| MOL002883 | Ethyl oleate (NF) | Paeoniae Radix Rubra | 310.58 | 32.4 | 1.4 | 0.19 |
| MOL002714 | baicalein | Paeoniae Radix Rubra | 270.25 | 33.52 | 0.63 | 0.21 |
| MOL007022 | evofolinB | Paeoniae Radix Rubra | 318.35 | 64.74 | 0 | 0.22 |
| MOL000492 | (+)-catechin | Paeoniae Radix Rubra | 290.29 | 54.83 | -0.03 | 0.24 |
| MOL013129 | (+)-3,3',5',5,7-Pentahydroflavanone | Paeoniae Radix Rubra | 304.27 | 63.17 | -0.34 | 0.27 |
| MOL006994 | 1-o-beta-d-glucopyranosyl-8-o-benzoylpaeonisuffrone_qt | Paeoniae Radix Rubra | 302.35 | 36.01 | -0.03 | 0.3 |
| MOL007018 | 9-ethyl-neo-paeoniaflorin A_qt | Paeoniae Radix Rubra | 334.4 | 64.42 | -0.01 | 0.3 |
| MOL006992 | (2R,3R)-4-methoxyl-distylin | Paeoniae Radix Rubra | 318.3 | 59.98 | 0.17 | 0.3 |
| MOL007005 | Albiflorin_qt | Paeoniae Radix Rubra | 318.35 | 48.7 | -0.38 | 0.33 |
| MOL006996 | 1-o-beta-d-glucopyranosylpaeonisuffrone_qt | Paeoniae Radix Rubra | 332.38 | 65.08 | -0.05 | 0.35 |
| MOL001918 | paeoniflorgenone | Paeoniae Radix Rubra | 318.35 | 87.59 | -0.09 | 0.37 |
| MOL001925 | paeoniflorin_qt | Paeoniae Radix Rubra | 318.35 | 68.18 | -0.34 | 0.4 |
| MOL007012 | 4-o-methyl-paeoniflorin_qt | Paeoniae Radix Rubra | 332.38 | 56.7 | 0.4 | 0.43 |
| MOL007008 | 4-ethyl-paeoniflorin_qt | Paeoniae Radix Rubra | 332.38 | 56.87 | -0.17 | 0.44 |
| MOL005043 | campest-5-en-3beta-ol | Paeoniae Radix Rubra | 400.76 | 37.58 | 1.32 | 0.71 |
| MOL002776 | Baicalin | Paeoniae Radix Rubra | 446.39 | 40.12 | -0.85 | 0.75 |
| MOL000358 | beta-sitosterol | Paeoniae Radix Rubra | 414.79 | 36.91 | 1.32 | 0.75 |
| MOL006999 | stigmast-7-en-3-ol | Paeoniae Radix Rubra | 414.79 | 37.42 | 1.32 | 0.75 |
| MOL004355 | Spinasterol | Paeoniae Radix Rubra | 412.77 | 42.98 | 1.44 | 0.76 |
| MOL000449 | Stigmasterol | Paeoniae Radix Rubra | 412.77 | 43.83 | 1.44 | 0.76 |
| MOL007004 | Albiflorin | Paeoniae Radix Rubra | 480.51 | 30.25 | -1.52 | 0.77 |
| MOL001924 | paeoniflorin | Paeoniae Radix Rubra | 480.51 | 53.87 | -1.47 | 0.79 |
| MOL001921 | Lactiflorin | Paeoniae Radix Rubra | 462.49 | 49.12 | -1.13 | 0.8 |
| MOL008457 | Tetrahydroalstonine | Paeoniae Radix Rubra | 352.47 | 32.42 | 0.9 | 0.81 |
| MOL001371 | Populoside_qt | Persicae Semen | 286.3 | 108.89 | 0.49 | 0.2 |
| MOL001340 | GA120 | Persicae Semen | 314.41 | 84.85 | 0.38 | 0.45 |
| MOL001339 | GA119 | Persicae Semen | 332.43 | 76.36 | -0.12 | 0.49 |
| MOL001328 | 2,3-didehydro GA70 | Persicae Semen | 330.41 | 63.29 | -0.27 | 0.5 |
| MOL001358 | gibberellin 7 | Persicae Semen | 330.41 | 73.8 | -0.18 | 0.5 |
| MOL001343 | GA122 | Persicae Semen | 330.41 | 64.79 | -0.17 | 0.5 |
| MOL001342 | GA121-isolactone | Persicae Semen | 330.41 | 72.7 | -0.26 | 0.54 |
| MOL001344 | GA122-isolactone | Persicae Semen | 330.41 | 88.11 | -0.18 | 0.54 |
| MOL001351 | Gibberellin A44 | Persicae Semen | 346.46 | 101.61 | -0.13 | 0.54 |
| MOL001320 | Amygdalin | Persicae Semen | 457.48 | 4.42 | -1.91 | 0.61 |
| MOL000493 | campesterol | Persicae Semen | 400.76 | 37.58 | 1.31 | 0.71 |
| MOL000296 | hederagenin | Persicae Semen | 414.79 | 36.91 | 1.32 | 0.75 |
| MOL000358 | beta-sitosterol | Persicae Semen | 414.79 | 36.91 | 1.32 | 0.75 |
| MOL001323 | Sitosterol alpha1 | Persicae Semen | 426.8 | 43.28 | 1.41 | 0.78 |
| MOL005448 | Leucine | Pheretima | 131.2 | 72.92 | -0.05 | 0.01 |
| MOL001443 | 4-Guanidino-1-butanol | Pheretima | 131.21 | 26.23 | 0.24 | 0.01 |
| MOL001831 | HYPOXANTHINE | Pheretima | 136.13 | 52.29 | 0.09 | 0.04 |
| MOL002687 | Guanosine | Pheretima | 283.28 | 21.43 | -1.21 | 0.21 |
| MOL002442 | Cholesteryl ferulate | Pheretima | 562.91 | 22.43 | 1.09 | 0.63 |

Table S2 The potential therapeutic targets of BYHWD against PH

| **Symbol** | **Name** | **Category** | **GeneCardsId** |
| --- | --- | --- | --- |
| MMP2 | Matrix Metallopeptidase 2 | Protein Coding | GC16P055390 |
| XDH | Xanthine Dehydrogenase | Protein Coding | GC02M031334 |
| PLAU | Plasminogen Activator, Urokinase | Protein Coding | GC10P073909 |
| FGFR1 | Fibroblast Growth Factor Receptor 1 | Protein Coding | GC08M038400 |
| MET | MET Proto-Oncogene, Receptor Tyrosine Kinase | Protein Coding | GC07P116672 |
| ALOX5 | Arachidonate 5-Lipoxygenase | Protein Coding | GC10P045374 |
| PLK1 | Polo Like Kinase 1 | Protein Coding | GC16P023933 |
| PTK2 | Protein Tyrosine Kinase 2 | Protein Coding | GC08M140657 |
| ADORA2A | Adenosine A2a Receptor | Protein Coding | GC22P024417 |
| PPARG | Peroxisome Proliferator Activated Receptor Gamma | Protein Coding | GC03P012287 |
| ABCB1 | ATP Binding Cassette Subfamily B Member 1 | Protein Coding | GC07M087504 |
| AHR | Aryl Hydrocarbon Receptor | Protein Coding | GC07P016916 |
| PIM1 | Pim-1 Proto-Oncogene, Serine/Threonine Kinase | Protein Coding | GC06P052164 |
| CYP19A1 | Cytochrome P450 Family 19 Subfamily A Member 1 | Protein Coding | GC15M051208 |
| MAPK14 | Mitogen-Activated Protein Kinase 14 | Protein Coding | GC06P052142 |
| CDK6 | Cyclin Dependent Kinase 6 | Protein Coding | GC07M092604 |
| CMA1 | Chymase 1 | Protein Coding | GC14M024506 |
| MPO | Myeloperoxidase | Protein Coding | GC17M058269 |
| SELP | Selectin P | Protein Coding | GC01M169558 |
| F2 | Coagulation Factor II, Thrombin | Protein Coding | GC11P046720 |
| CA9 | Carbonic Anhydrase 9 | Protein Coding | GC09P035673 |
| ARG1 | Arginase 1 | Protein Coding | GC06P131473 |
| NPR3 | Natriuretic Peptide Receptor 3 | Protein Coding | GC05P032689 |
| TGFBR1 | Transforming Growth Factor Beta Receptor 1 | Protein Coding | GC09P099104 |
| NOX4 | NADPH Oxidase 4 | Protein Coding | GC11M089324 |
| BMP2 | Bone Morphogenetic Protein 2 | Protein Coding | GC20P006696 |
| GBA | Glucosylceramidase Beta | Protein Coding | GC01M155234 |
| PPIA | Peptidylprolyl Isomerase A | Protein Coding | GC07P044807 |
| ANG | Angiogenin | Protein Coding | GC14P020872 |
| CTSD | Cathepsin D | Protein Coding | GC11M001752 |
| ACHE | Acetylcholinesterase (Cartwright Blood Group) | Protein Coding | GC07M100889 |
| CASP3 | Caspase 3 | Protein Coding | GC04M184627 |
| HSP90AA1 | Heat Shock Protein 90 Alpha Family Class A Member 1 | Protein Coding | GC14M102080 |
| PARP1 | Poly(ADP-Ribose) Polymerase 1 | Protein Coding | GC01M226360 |
| HMGCR | 3-Hydroxy-3-Methylglutaryl-CoA Reductase | Protein Coding | GC05P075336 |
| PPARD | Peroxisome Proliferator Activated Receptor Delta | Protein Coding | GC06P052126 |
| GSR | Glutathione-Disulfide Reductase | Protein Coding | GC08M030678 |
| KDR | Kinase Insert Domain Receptor | Protein Coding | GC04M055078 |
| ABCG2 | ATP Binding Cassette Subfamily G Member 2 (Junior Blood Group) | Protein Coding | GC04M088090 |
| ALOX12 | Arachidonate 12-Lipoxygenase, 12S Type | Protein Coding | GC17P006995 |
| MMP3 | Matrix Metallopeptidase 3 | Protein Coding | GC11M102835 |
| CXCR1 | C-X-C Motif Chemokine Receptor 1 | Protein Coding | GC02M218162 |
| MAPK1 | Mitogen-Activated Protein Kinase 1 | Protein Coding | GC22M021754 |
| PIK3CG | Phosphatidylinositol-4,5-Bisphosphate 3-Kinase Catalytic Subunit Gamma | Protein Coding | GC07P106865 |
| EGFR | Epidermal Growth Factor Receptor | Protein Coding | GC07P055019 |
| IL2 | Interleukin 2 | Protein Coding | GC04M122451 |
| TOP2A | DNA Topoisomerase II Alpha | Protein Coding | GC17M040388 |
| MAPK8 | Mitogen-Activated Protein Kinase 8 | Protein Coding | GC10P048306 |
| IGF1R | Insulin Like Growth Factor 1 Receptor | Protein Coding | GC15P098648 |
| F10 | Coagulation Factor X | Protein Coding | GC13P113122 |
| AKR1B1 | Aldo-Keto Reductase Family 1 Member B | Protein Coding | GC07M134442 |
| NOS3 | Nitric Oxide Synthase 3 | Protein Coding | GC07P150990 |
| SRC | SRC Proto-Oncogene, Non-Receptor Tyrosine Kinase | Protein Coding | GC20P037344 |
| CDK2 | Cyclin Dependent Kinase 2 | Protein Coding | GC12P055966 |
| PLG | Plasminogen | Protein Coding | GC06P160702 |
| TGFBR2 | Transforming Growth Factor Beta Receptor 2 | Protein Coding | GC03P030623 |
| AKT1 | AKT Serine/Threonine Kinase 1 | Protein Coding | GC14M104769 |
| LCN2 | Lipocalin 2 | Protein Coding | GC09P128149 |
| SPARC | Secreted Protein Acidic And Cysteine Rich | Protein Coding | GC05M151661 |
| ALB | Albumin | Protein Coding | GC04P073397 |
| AR | Androgen Receptor | Protein Coding | GC0XP067544 |
| ESR2 | Estrogen Receptor 2 | Protein Coding | GC14M064084 |
| EPHB4 | EPH Receptor B4 | Protein Coding | GC07M100803 |
| CYP1B1 | Cytochrome P450 Family 1 Subfamily B Member 1 | Protein Coding | GC02M038066 |
| MMP9 | Matrix Metallopeptidase 9 | Protein Coding | GC20P046008 |

Table S3 The result of top 20 GO (biological process) enrichment analysis

| **Category** | **Term** | **P-Value** | **Benjamini** |
| --- | --- | --- | --- |
| GOTERM_BP_DIRECT | GO:0043066~negative regulation of apoptotic process | 1.17E-10 | 1.46E-07 |
| GOTERM_BP_DIRECT | GO:0018105~peptidyl-serine phosphorylation | 2.35E-08 | 1.47E-05 |
| GOTERM_BP_DIRECT | GO:0030335~positive regulation of cell migration | 3.07E-08 | 1.28E-05 |
| GOTERM_BP_DIRECT | GO:0001525~angiogenesis | 1.60E-07 | 4.99E-05 |
| GOTERM_BP_DIRECT | GO:0008284~positive regulation of cell proliferation | 1.61E-07 | 4.02E-05 |
| GOTERM_BP_DIRECT | GO:0042493~response to drug | 2.08E-07 | 4.34E-05 |
| GOTERM_BP_DIRECT | GO:0046777~protein autophosphorylation | 2.80E-07 | 4.99E-05 |
| GOTERM_BP_DIRECT | GO:0010628~positive regulation of gene expression | 6.22E-07 | 9.72E-05 |
| GOTERM_BP_DIRECT | GO:0051897~positive regulation of protein kinase B signaling | 7.77E-07 | 1.08E-04 |
| GOTERM_BP_DIRECT | GO:0018108~peptidyl-tyrosine phosphorylation | 1.83E-06 | 2.28E-04 |
| GOTERM_BP_DIRECT | GO:0048661~positive regulation of smooth muscle cell proliferation | 3.19E-06 | 3.62E-04 |
| GOTERM_BP_DIRECT | GO:0001666~response to hypoxia | 3.98E-06 | 4.14E-04 |
| GOTERM_BP_DIRECT | GO:0014068~positive regulation of phosphatidylinositol 3-kinase signaling | 4.75E-06 | 4.57E-04 |
| GOTERM_BP_DIRECT | GO:2000379~positive regulation of reactive oxygen species metabolic process | 4.88E-06 | 4.36E-04 |
| GOTERM_BP_DIRECT | GO:0001938~positive regulation of endothelial cell proliferation | 6.39E-06 | 5.32E-04 |
| GOTERM_BP_DIRECT | GO:0006468~protein phosphorylation | 8.05E-06 | 6.29E-04 |
| GOTERM_BP_DIRECT | GO:0001934~positive regulation of protein phosphorylation | 8.70E-06 | 6.40E-04 |
| GOTERM_BP_DIRECT | GO:0018107~peptidyl-threonine phosphorylation | 1.28E-05 | 8.92E-04 |
| GOTERM_BP_DIRECT | GO:0042060~wound healing | 1.32E-05 | 8.70E-04 |
| GOTERM_BP_DIRECT | GO:0030307~positive regulation of cell growth | 1.68E-05 | 0.001049 |

Table S4 The result of top 20 GO (molecular function) enrichment analysis

| **Category** | **Term** | **P-Value** | **Benjamini** |
| --- | --- | --- | --- |
| GOTERM_MF_DIRECT | GO:0005524~ATP binding | 1.25E-08 | 3.64E-06 |
| GOTERM_MF_DIRECT | GO:0019899~enzyme binding | 4.59E-07 | 6.70E-05 |
| GOTERM_MF_DIRECT | GO:0004713~protein tyrosine kinase activity | 6.87E-07 | 6.69E-05 |
| GOTERM_MF_DIRECT | GO:0005515~protein binding | 4.16E-06 | 3.04E-04 |
| GOTERM_MF_DIRECT | GO:0004252~serine-type endopeptidase activity | 5.18E-06 | 3.02E-04 |
| GOTERM_MF_DIRECT | GO:0004672~protein kinase activity | 7.91E-06 | 3.85E-04 |
| GOTERM_MF_DIRECT | GO:0004879~RNA polymerase II transcription factor activity, ligand-activated sequence-specific DNA binding | 1.01E-05 | 4.21E-04 |
| GOTERM_MF_DIRECT | GO:0004714~transmembrane receptor protein tyrosine kinase activity | 1.26E-05 | 4.59E-04 |
| GOTERM_MF_DIRECT | GO:0005506~iron ion binding | 2.44E-05 | 7.92E-04 |
| GOTERM_MF_DIRECT | GO:0016301~kinase activity | 3.44E-05 | 0.001005 |
| GOTERM_MF_DIRECT | GO:0004674~protein serine/threonine kinase activity | 8.38E-05 | 0.002222 |
| GOTERM_MF_DIRECT | GO:0019903~protein phosphatase binding | 9.46E-05 | 0.0023 |
| GOTERM_MF_DIRECT | GO:0020037~heme binding | 1.70E-04 | 0.003809 |
| GOTERM_MF_DIRECT | GO:0005102~receptor binding | 3.68E-04 | 0.007641 |
| GOTERM_MF_DIRECT | GO:0009055~electron carrier activity | 3.76E-04 | 0.007294 |
| GOTERM_MF_DIRECT | GO:0030235~nitric-oxide synthase regulator activity | 3.90E-04 | 0.007101 |
| GOTERM_MF_DIRECT | GO:0042802~identical protein binding | 4.80E-04 | 0.008221 |
| GOTERM_MF_DIRECT | GO:0008134~transcription factor binding | 7.12E-04 | 0.011481 |
| GOTERM_MF_DIRECT | GO:0003707~steroid hormone receptor activity | 0.001248 | 0.019014 |
| GOTERM_MF_DIRECT | GO:0004707~MAP kinase activity | 0.00125 | 0.018101 |
